# D-Tagatose Is a Promising Sweetener to Control Glycaemia: A New Functional Food

**DOI:** 10.1155/2018/8718053

**Published:** 2018-01-09

**Authors:** Marion Guerrero-Wyss, Samuel Durán Agüero, Lisse Angarita Dávila

**Affiliations:** ^1^Escuela de Nutrición, Facultad Ciencias De La Salud, Universidad San Sebastián, Santiago, Chile; ^2^Carrera de Nutrición, Facultad de Medicina, Universidad Andres Bello, Sede Concepción, Talcahuano, Chile

## Abstract

The objective of the current research was to review and update evidence on the dietary effect of the consumption of tagatose in type 2 diabetes, as well as to elucidate the current approach that exists on its production and biotechnological utility in functional food for diabetics. Articles published before July 1, 2017, were included in the databases PubMed, EBSCO, Google Scholar, and Scielo, including the terms “Tagatose”, “Sweeteners”, “Diabetes Mellitus type 2”, “Sweeteners”, “D-Tag”. D-Tagatose (D-tag) is an isomer of fructose which is approximately 90% sweeter than sucrose. Preliminary studies in animals and preclinical studies showed that D-tag decreased glucose levels, which generated great interest in the scientific community. Recent studies indicate that tagatose has low glycemic index, a potent hypoglycemic effect, and eventually could be associated with important benefits for the treatment of obesity. The authors concluded that D-tag is promising as a sweetener without major adverse effects observed in these clinical studies.

## 1. Introduction

D-Tagatose (D-tag) is an isomer of fructose which is approximately 90% sweeter than sucrose. Fructose that corresponds to a natural hexose was developed as a low-calorie sugar substitute.

Only 20% of the orally ingested tagatose is metabolized completely and mainly in the liver [1]. In 2001, D-tag was appointed by the Food and Drug Administration (FDA) as a generally recognized safe product (GRAS), and subsequently it has been used as a nutritional sweetener or low in calories [2]. After this, the European Union (EU) introduced D-tag as a “new food ingredient,” without any restrictions on the amount to be used [1, 2].

Currently, D-tag is used as a sweetener in beverages, yogurt, creams, and dietetic candy [3].

A method for the mature production of D-tag is the direct isomerization of G-galactose in D-tag, with metal hydroxides such as chemical catalysts in basic conditions [4].

Preliminary animal studies and preclinical studies showed that D-tag decreased glucose levels, generating great interest in the scientific community [5]. The proposed action mechanism may involve interference in the absorption of carbohydrates through inhibition of intestinal disaccharidases and glucose transportation. It can also act through the inhibition of hepatic glycogenolysis [1]. In addition to presenting an effect in the reduction of total cholesterol, VLDL, and LDL compared with sucrose [6], likewise D-tag has contributed to increasing levels of HDL cholesterol [7].

The D-tag would have an antihyperglycemic potential through its beneficial effects on the increment of postprandial serum glucose and hyperinsulinemia. Recent studies indicate that tagatose has a powerful antidiabetic effect and could eventually be associated with important benefits for the treatment of obesity. However, preliminary results of a study indicated that there were not any changes in glucose or insulin levels after oral administration of D-tag while fasting [8].

The objective of this research is to review and update evidence about the therapeutic effect of the consumption of tagatose in patients with diabetes type 2 as well as to elucidate the current approach that exists on its production and biotechnological use in functional food for diabetics.

## 2. Methodology

Articles published before July 1, 2017, were selected for the completion of this review; the search was carried out in the databases PubMed, EBSCO, Google Scholar, and Scielo, including terms or key words “Tagatose”, “Sweeteners”, “Diabetes Mellitus type 2”, “Sweeteners”, “D-Tag”.

This review included clinical interventions in animals and humans, as well as studies on the formulation and production of food that contain tagatose. The search included English and Spanish as languages.

## 3. Tagatose and Blood Glucose

A phase II study with more than 6 months of duration and a phase III effectiveness study with more than 12 months of duration used D-tag to reduce HbA1c in 161 and 494 diabetic patients, respectively, in the United States and India. The results showed a statistically significant reduction in HbA1c compared to the placebo group, concluding that it could eventually become a treatment for diabetes [10].

A cross, randomized, double-blind experimental design determined the supplementation effect with tagatose on postprandial hyperglycemia in 85 Korean hyperglycemic individuals (*n* = 52 and *n* = 33) [1] (Table 1). Blood samples were taken during fasting and after drinking a beverage with sucralose-erythritol (placebo) and other formulation with tagatose at 0, 30, 60, and 120 minutes, analyzing glucose, insulin, C-peptide, and lipid profile [1]. Hyperglycemic individuals of legal age had higher levels of triglycerides, Col-total, LDL-Col, A1, and B apolipoprotein. After the intake of a beverage with tagatose (5 g), only hyperglycemic individuals had a significant reduction of blood glucose at 120 min (*P* = 0.019), as well as in the blood glucose area under the curve (AUC) (*P* = 0.017) [1]. Normal individuals who received a high dose of the beverage with tagatose (10 g) showed decreased levels of serum insulin, AUC of insulin, and C-peptide. Therefore, these results suggest that a beverage sweetened with tagatose could control the postprandial glycemic response in individuals with hyperglycemia [1].

Another study [11] was performed to investigate acute effects on blood sugar levels in 8 healthy individuals and in 8 individuals with DM 2, after the oral intake of 75 g of D-tagatose (D-tag) alone and combined with 75 g of glucose. Diabetics received separately and at 0, 10, 15, 20, and 30 min 75 g of D-tag, 30 minutes before a dose of 75 g of glucose. Oral load with D-tag did not alter blood sugar levels or insulin in any group. The pretreatment with 75 g of D-tag attenuated the glycemic curve in diabetics [11], significantly reducing the blood glucose area under the curve (AUC) and the glycemic increment after the ingestion of glycoside solution [11]. Gastrointestinal adverse effects caused by high doses of D-tag suggest that this may act to reduce intestinal glucose absorption.

In another randomized research [12] whose main objective was to evaluate the safety and effect of D-tag on the glycemic control in patients with DM 2 according to levels of glycosylated hemoglobin (HbA1c) at the end of 6 months with different doses of D-tag, 2.5 g, 5.0 g, or 7.5 g (3 times/day), controlling the serum level of blood glucose, plasma lipids, HbA1c, changes in body weight, and body mass index, as well as insulin variations [12], basal glycaemia dropped in 3 and 6 months just in the group that received a dose of 7.5 g. The average body weight dropped directly in greater proportion to doses of 5.0 g and 7.5 g of D-tag. The minimum amount necessary to reduce the HbA1c corresponded to 5.0 g, while the highest dose (7.5 g) provided a greater effect on the evaluated parameters [12].

### 3.1. Proposed Mechanisms of the Metabolic Effect of D-Tag

**Figure 1:**
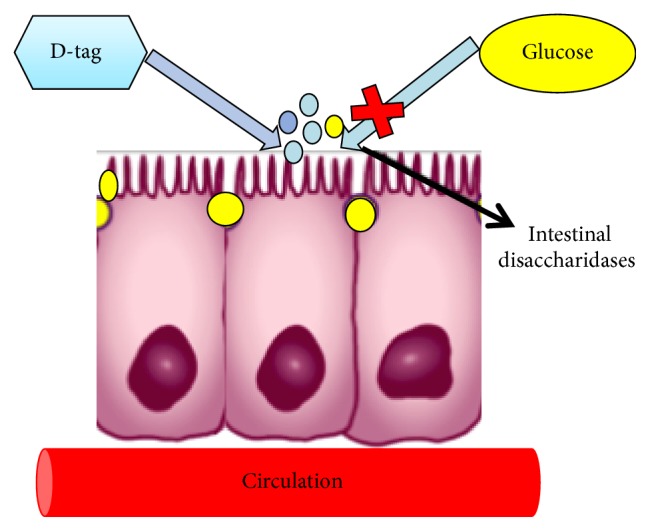
D-Tagatose glycemic control; proposed mechanism explained that D-tagatose directly inhibits the absorption of glucose by intestinal disaccharidases.* Note*. Marion Guerrero-Wyss, Samuel Durán Agüero, Lisse Angarita Dávila, 2017.

It should be noted that the mechanisms related to the effect of D-tagatose on the regulation of glycaemia are still under study. However, some authors have described various mechanisms by which the antihyperglycemic effect of D-tagatose could be explained, highlighting a direct inhibition of intestinal disaccharidases, which would increase as the period of intestinal exposure increases to D-tagatose (Figure 1).

**Figure 2:**
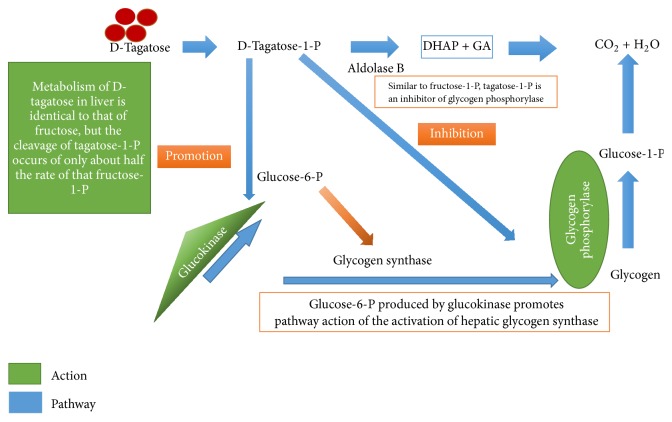
D-Tagatose glycemic control; proposed mechanism explained through the inhibition of hepatic glycogenolysis.* Note*. Adapted from the original “Tagatose Glycemic Control MOA in the Liver” produced by Muddada, 2012 [13].

Another proposed hypothesis could be explained through the inhibition of hepatic glycogenolysis [1] (Figure 2). Tagatose seems to act by promoting glycogen synthesis and to decrease glycogen utilization. Sudhamani Muddada explains that D-tag competitively inhibits the enzyme that metabolizes glycogen [13], causing glucose to remain stored as glycogen. On the other hand, it promotes the metabolism of glucose to glucose-6-P, which stimulates storage of glucose as glycogen. The intermediate of tagatose metabolism tagatose-1-phosphate promotes the activity of glucokinase, resulting in increased phosphorylation of glucose to glucose-1-phosphate which activates glycogen synthase mobilising glucose to glycogen. Tagatose-1-phosphate inhibits the activity of glycogen phosphorylase preventing glycogen breakdown. It is hypothesised that tagatose is metabolized like fructose but at a slower rate [13]. Also, tagatose prevents absorption of sucrose and maltose by inhibiting the action of sucrases and maltases in the small intestine [1].

On the other hand, the interaction of ingested nutrients with the small intestine to stimulate the release of gut peptides, including glucagon-like peptide 1 (GLP-1) and glucose-dependent insulinotropic polypeptide (GIP), represents a major mechanism in the regulation of gastric emptying, satiety, and insulin secretion [11]. In this sense, the capacity of tagatose to increase GLP-1 secretion is highly relevant to the lowering of blood glucose. D-tag is reported to stimulate GLP-1 to a comparable degree to fructose, whereas it did not stimulate GIP [11]. Because neither fructose nor tagatose is sodium-glucose cotransporter-1 (SGLT1) substrate, it follows that signaling pathways other than SGLT1 are likely to be involved in GLP-1 secretion. The exposure of poorly absorbed sugars to the distal gut, with the production of short-chain fatty acids by bacterial fermentation, may represent an important mechanism of GLP-1 stimulation [14]. Xylose, a poorly absorbed pentose, is a potent stimulus for GLP-1, and tagatose may act in a similar manner, given its relatively low absorption rate (~25%). Wu et al. [15] showed that, in healthy humans, the preloads with tagatose/isomaltose (TIM), partially absorbed, promoted a prolonged secretion of GLP-1; this is probably stimulated by a long length in gut and therefore results in later GLP-1 secretion but does not stimulate GIP. Nevertheless, GLP-1 was stimulated to a greater level by the TIM than bythe sucralose preload immediately after the meal, and tagatose was also shown to slow gastric emptying rapidly [15], so that early gastrointestinal responses to tagatose might be mediated by other pathways, such as GLUT5 (the fructose transporter) [15].

On the other hand, a positive effect of D-tag in the reduction of total cholesterol, VLDL, and LDL compared with sucrose has been reported [6]. The proposed action mechanism may involve reduced pyruvate generation from glycolysis, reducing acetyl CoA through the Krebs cycle as a precursor to cholesterol [13]. Some authors have also described that D-tag has contributed to increasing levels of HDL cholesterol [7]. Recently, it was suggested that D-tagatose blocks absorption of fructose through the gut and can effectively reduce diet-induced dyslipidemia [13].

## 4. Toxicity of D-Tagatose

A study was conducted to detect toxicity of D-tag in rats (SD) [16], administering D-tag in three doses (4,000, 12,000, and 20,000 mg/kg body weight/day) through gastric intubation on days 6 to 15 of gestation period. Related toxicity or clinical effects associated in maternal rats at a dose of 4000 mg/kg/day were not observed. In the mid and high doses, nonformed stools were observed; this effect was more prominent in the early period of treatment (6th to 8th day) attributed to the osmotic action of the big quantity administered of D-tag. This molecule is not well digested or absorbed; most part of the sugar goes to the colon, where it absorbs water and is fermented by colonic bacteria. The group submitted to the highest dose experienced an average weight loss during the interval from the 6th to the 9th day of gestation, considered as a direct result of the laxative effect. In addition to this action, the reduced consumption of food also contributed to the decrease in weight gain. Animals exposed to medium and high dose presented one food intake less than the control group. Adverse effects on reproductive performance were not observed in treatment groups or in the fetus overweight, neither in the distribution by sex, weight of the liver, nor external, visceral, or skeletal malformations in any dose [16].

The potential genotoxic effect of this molecule was examined in five standard trials [17]. In these tests, there was not any significant increment in the ovary cells of Chinese hamster with chromosomal alterations in concentrations up to 5000 mcg/ml with or without metabolic activation. It was not found that D-tag could increase the frequency of lymphoma cancer cells of mouse L5178Y with or without metabolic activation; additionally, D-tag did not modify micronucleus of polychromatic erythrocytes in the bone marrow, concluding that this molecule was not genotoxic in the trials described above [17].

Another clinical, cross, and double-blind study evaluated the effect of this monosaccharide against D-fructose on the increment in the production of uric acid [18], through the acceleration of purine nucleotide degradation as well as other metabolic parameters in 8 male individuals. It was detected that both the highest concentration of uric acid and the 4-hour AUC were significantly higher after the intake of D-tag compared to 30 g D-fructose or water. It is concluded that D-tag attenuates glycemic and insulin response of a food 255 minutes after its ingestion. In addition, both sweeteners increased cholecystokinin (CCK) and peptide levels similar to glucagon-1 (GLP-1).

In the same two-phase investigation mentioned above [18] carried out in 8 healthy individuals and in 8 individuals with diabetes type 2, the effect of repeated doses of D-tag in urea, phosphorus, magnesium, lipids, and glycemic homeostasis was determined. In the first phase, 75 g of glucose was administered and oral tolerance of glucose and D-tag test was assessed. Uric acid, phosphorus, and magnesium levels were determined in blood samples collected at 0, 30, 60, 120, and 180 minutes after the intake. Healthy individuals received at random 75 g of D-tag or sucrose (25 g with each meal) per day for 8 weeks. Diabetics were assigned into two groups and received 75 g of D-tag or a supplement without sugar every day for 8 weeks. This test did not show a basal increment of uric acid in response to the daily intake of D-tag. However, a transient increase in the plasma level of uric acid was observed after the single dose of 75 g of D-tag in the tolerance test at 60 minutes. In accordance with the previous observations on the fructose, the increment of uric acid in plasma was associated with a slight decrease of phosphorus in plasma and a slight increase of magnesium. The daily intake of D-tag for 8 weeks did not have any effect on the magnesium in plasma in fasting, phosphorous, cholesterol, triglycerides, HbA1c, glucose, and insulin. Ingestion of three doses of 25 g/day for 8 weeks resulted in symptoms of flatulence in seven of the eight individuals and some diarrhea in six individuals. The authors concluded that D-tag is a promising sweetener without adverse effects observed in these trials.

Several studies were conducted to evaluate the gastrointestinal symptoms in humans; these were analyzed after the consumption of 29 or 30 g of D-tag [19]. Nausea and diarrhea were reported with an incidence of 15.1 and 31.5%, respectively, in 73 healthy young men. The increase in flatulence after D-tagatose was frequently reported in all studies and did not diminish over a period of 15 days with the intake of 30 g in a single dose daily. In most of the cases, moderate symptoms were reported. However, the results suggest that the dose of 30 g ingested at a specific time can be superior to the dose that should be recommended for ordinary use.

Another study compared the effect of carbohydrate in the form of sucrose and D-tag on the plasma concentrations of cholesterol, hyperglycemia, and atherosclerosis [6]. Mice of both genders were fed with standard diet or a diet enriched with sucrose or D-tag as a source of carbohydrates for 16 weeks; both diets contained equivalent amounts (g/kg) of macronutrients. The intake of food, body weight, and diameter of adipocytes, concentration of cholesterol and serum lipoproteins, and aortic atherosclerosis were evaluated. Immune-staining of macrophages and the contents of collagen in the aortic root lesions were examined.

Mice fed with D-tag showed similar intake of energy, body weight, blood sugar, and insulin, but the group fed with sucrose exhibited a higher energy intake, obesity, and hyperglycemia. There was an increment in the diameter of adipocytes, the levels of cholesterol and plasma triglycerides, atherosclerosis, immune-staining in macrophages, and reduction in collagen content compared to mice that consumed D-tag and the control group. These results show that compared with sucrose the equivalent substitution of D-tag as a carbohydrate in the diet does not lead to the same proportion of obesity, hyperglycemia, hyperlipidemia, and atherosclerosis [6].

A pilot study explored metabolic effects of D-tag [7] administered orally at a dose of 15 g of D-tag 3 times a day with food, in individuals with diabetes type 2 for 12 months. None of the serious adverse effects were observed during the treatment; 10 out of the 12 individuals recruited initially experienced gastrointestinal side effects that tended to be mild and transient. Average body weight decreased from 108.4 kg to 103.3 kg (*P* = 0.001). HbA1c did not have a significant reduction; it went from 10.6% to 9.6% (*P* = 0.08). HDL cholesterol levels increased progressively from a base level of 30.5 to 41.7 mg/dl in the 12 months in 6 individuals who did not use lipid-modifying drugs during the study (*P* < 0.001). In conclusion, D-tag improved body weight and HDL cholesterol in this pilot study.

Another trial [9] compared the effects of groups drug (D-tag) and placebo (sucralose). The dose of D-tag was 15 g dissolved in 125 to 250 ml of water three times a day (TD); the placebo dose was 1.5 g dissolved in 125 to 250 ml of water TD. The authors concluded that D-tag was effective in reducing the level of HbA1c when it is administered for two months at a dose of 15 g three times a day just before meals [9].

## 5. Glycemic Index and Glycemic Load of Tagatose

Several studies have shown a positive effect after the intake of tagatose in healthy individuals [9–11, 20] resulting in a low GI [20], compared to glucose and white bread as a reference food, glycemic index of D-tagatose was 3 and 4, respectively. While investigating the GI of other sweeteners like maltitol (=26), xylitol (=8), or isomaltulose (=32) [21], it is observed that D-tag has less value even when compared to sweetener blends composed of polydextrose and sorbitol (=7) [20]. On the other hand, there is evidence of a null or very low glycemic load GL (=0), compared to the value reported for maltitol (GL = 3), xylitol (=1), isomaltulose (=3), or the mixture of sorbitol and polydextrose (=1) [20]. It is known that the indicators of GI and GL should be done initially in healthy individuals to obtain a metabolic reference compared with diabetic individuals [22]. Recognizing that the glycemic variability is very high [23] and specific according to the type of product or food [24], these studies should be extended with more frequency in patients with diabetes type 2 in order to compare differences in the stability of the glycemic curve and get the value of these indicators in individuals with this type of pathology [25, 26].

## 6. New Approaches in the Production and Biotechnology Usefulness of Tagatose

The potential applications of this monosaccharide in the pharmaceutical industry and the agri-food market have reached a boom [27]. However, the use of D-tag is limited by its high production cost [28]. Compared to the intracellular enzymes, extracellular route is an interesting strategy to increase the adequacy of biocatalysts [26]. Numerous studies [28–31] have focused on this objective; recently a gene (TM0416) encoding protein D-tag 3-epimerase from a hyperthermophile marine bacterium has been studied; this metalloenzyme showed an unusual high activity for the epimerization of D-tag to D-sorbose, advance that could be functionally classified in the production of unusual sugars [30] and an alternative to produce vitamin C [28, 30].

Regarding its healthy prebiotic effect, it is known that the chemical structure of tagatose must not be altered during the processing and storage of food [27]. In this regard, several studies have evaluated the thermal stability of this sweetener in milk and lemonade in different concentrations, concluding that this monosaccharide can be used in the formulation of drinks for people with diabetes with a minimum chance of degradation and very low loss of prebiotic activity [32, 33].

Another recent investigation [34] highlighted the potential of the* Lb. casei* to reduce the accumulation of galactose in fermented milk by the metabolic pathway tagatose-6-phosphate (T6-P) specific for this species; this process, facing the residual difficulties caused by lactose and galactose in fermented dairy food, would be a potential alternative. These wonderful advances in food technology make this molecule an ideal sweetener in functional products for patients with diabetes [26, 32, 33], with the ability to positively affect the intestinal microbiota of these patients, making its consumption more interesting and useful in a little explored area [34–42].

## 7. Conclusion

After a decade of studies, tagatose became generally recognized as a safe product to be used in food and beverages under FDA Regulation of The United States. A subsequent trial that lasted 14 months confirmed its potential use to treat type 2 diabetes, showing great promise for inducing weight loss and increased cholesterol of high density lipoproteins, as well as its importance for the control of diabetes. There are no current therapies for type 2 diabetes that provide these benefits. Several studies indicate that predominant side effects of tagatose are gastrointestinal disorders only associated with excessive consumption, effects that do not exceed a specific period of 2 weeks, and these have been only observed after the consumption of high doses of tagatose.

## Figures and Tables

**Table 1:** Antihyperglycemic effect of D-tagatose.

Sample population	Methodology	Findings	Mechanism described or proposed
Normal subjects and type 2 diabetics patients.	They were given 75 g of glucose, 75 g of D-tag, or 75 g of D-tag 30 min prior to a 75 g oral glucose tolerance test.	The glucose area under the curve (AUC) was reduced significantly also by pretreatment with D-tag in a dose-dependent manner in patients with diabetes mellitus (*P* < 0.05 for 10 g D-tag, *P* < 0.001 for 20 g D-tag, and *P* = 0.0001 for 30 g D-tag) [8].	D-Tagatose directly inhibits the absorption of glucose by intestinal disaccharidases.

Type 2 diabetics patients.	They were given D-tag in three treatment groups: 2.5 g, 5.0 g, and 7.5 g given orally (three times daily, immediately prior to meals). Eight weeks after screening and stabilization of diabetics.	Only the 7.5 g dosage group exhibited reductions of fasting glucose from baseline at the 3- and 6-month time points [1].	Inhibition of sucrose activity by D-tag has been in rabbit small intestine.

Type 2 diabetics patients.	Two randomized groups were given a dose of D-tag (15 g) and the other group was given a dose of placebo (1.5 g), which were dissolved in 125 to 250 ml of water three times a day (TD).	D-Tagatose significantly reduced HbA1c compared to placebo. D-Tagatose was effective at reducing the HbA1c level when administered for two months at doses of 15 g TID. Also significant reductions in the HbA1c level at six and ten months were also met [9].	Unlike many other diabetes drugs, the longer the D-tagatose therapy, the better the efficacy, since the intestinal mucosa will be exposed for a longer period to D-tag.

## References

[1] Lu Y., Levin G. V., Donner T. W. (2008). Tagatose, a new antidiabetic and obesity control drug. *Diabetes, Obesity and Metabolism*.

[2] Levin G. V. (2002). Tagatose, the new GRAS sweetener and health product. *Journal of Medicinal Food*.

[3] Rulis Agency response letter GRAS notice, http://www.fda.gov/Food/IngredientsPackagingLabeling/GRAS/NoticeInventory/ucm245241.htm

[4] Kim P. (2004). Current studies on biological tagatose production using L-arabinose isomerase: A review and future perspective. *Applied Microbiology and Biotechnology*.

[5] Szepesi B., Levin G., Zehner L., Saunders J. (1996). Antidiabetic effect of D-tagatose in shr/n-cp rats. *The FASEB Journal*.

[6] Police S. B., Harris J. C., Lodder R. A., Cassis L. A. (2009). Effect of diets containing sucrose vs. D-tagatose in hypercholesterolemic mice. *Obesity*.

[7] Donner T. W., Magder L. S., Zarbalian K. (2010). Dietary supplementation with d-tagatose in subjects with type 2 diabetes leads to weight loss and raises high-density lipoprotein cholesterol. *Nutrition Research*.

[8] Donner T. W., Wilber J. F., Ostrowski D. (1996). D-tagatose: A novel therapeutic adjunct for non-insulin-dependent diabetes. *Diabetes*.

[9] Ensor M., Banfield A., Smith R., Williams J., Lodder R. (2015). Safety and efficacy of D-tagatose in glycemic control in subjects with type 2 diabetes. *Journal of Endocrinology, Diabetes & Obesity*.

[10] Fujisawa T., Riby J., Kretchmer N. (1991). Intestinal absorption of fructose in the rat. *Gastroenterology*.

[11] Donner T. W., Wilber J. F., Ostrowski D. (1999). D-tagatose, a novel hexose: Acute effects on carbohydrate tolerance in subjects with and without type 2 diabetes. *Diabetes, Obesity and Metabolism*.

[12] Ensor M., Williams J., Smith R., Banfield A., Lodder R. A. (2014). Effects of three low-doses of D-tagatose on glycemic control over six months. *Journal of Endocrinology, Diabetes & Obesity*.

[13] Muddada S. (2012). Tagatose: the multifunctional food ingredient and potential drug. *Journal of Pharmacy Research*.

[14] Wu T., Rayner C. K., Jones K., Horowitz M. (2010). Dietary Effects on Incretin Hormone Secretion. *Vitamins & Hormones*.

[15] Wu T., Zhao B. R., Bound M. J. (2012). Effects of different sweet preloads on incretin hormone secretion, gastric emptying, and postprandial glycemia in healthy humans. *American Journal of Clinical Nutrition*.

[16] Kruger C. L., Whittaker M. H., Frankos V. H., Schroeder R. E. (1999). Developmental toxicity study of D-tagatose in rats. *Regulatory Toxicology and Pharmacology*.

[17] Kruger C. L., Whittaker M. H., Frankos V. H. (1999). Genotoxicity tests on D-tagatose. *Regulatory Toxicology and Pharmacology*.

[18] Saunders J. P., Donner T. W., Sadler J. H., Levin G. V., Makris N. G. (1999). Effects of acute and repeated oral doses of D-tagatose on plasma uric acid in normal and diabetic humans. *Regulatory Toxicology and Pharmacology*.

[19] Buemann B., Toubro S., Raben A., Astrup A. (1999). Human tolerance to a single, high dose of D-tagatose. *Regulatory Toxicology and Pharmacology*.

[20] Atkinson F. S., Foster-Powell K., Brand-Miller J. C. (2008). International tables of glycemic index and glycemic load values: 2008. *Diabetes Care*.

[21] Holub I., Gostner A., Theis S. (2010). Novel findings on the metabolic effects of the low glycaemic carbohydrate isomaltulose (Palatinose™). *British Journal of Nutrition*.

[22] Augustin L. S. A., Kendall C. W. C., Jenkins D. J. A. (2015). Glycemic index, glycemic load and glycemic response: An International Scientific Consensus Summit from the International Carbohydrate Quality Consortium (ICQC). *Nutrition, Metabolism & Cardiovascular Diseases*.

[23] Matthan N. R., Ausman L. M., Meng H., Tighiouart H., Lichtenstein A. H. (2016). Estimating the reliability of glycemic index values and potential sources of methodological and biological variability. *American Journal of Clinical Nutrition*.

[24] Devitt A. A., Williams J. A., Choe Y. S., Hustead D. S., Mustad V. A. (2013). Glycemic responses to glycemia-targeted specialized-nutrition beverages with varying carbohydrates compared to a standard nutritional beverage in adults with type 2 diabetes. *Advances in Bioscience and Biotechnology*.

[25] Angarita L., López J., Aparicio D., Parra K., Uzcátegui M., Duran S. (2017). Índice glicémico, carga glicémica e insulina postprandial a dos fórmulas isoglucídicas con distintos edulcorantes y fibra en adultos sanos y diabéticos tipo 2. *Nutricion Hospitalaria*.

[26] Angarita Dávila L., Duran S., Aparicio Camargo D. (2017). Rol de la estevia y L- carnitina sobre el impacto glicémico de un suplemento nutricional en adultos. *Nutrición Hospitalaria*.

[27] Bell L. N., Luecke K. J. (2012). Tagatose stability in milk and diet lemonade. *Journal of Food Science*.

[28] Patel M. J., Akhani R. C., Patel A. T., Dedania S. R., Patel D. H. (2017). A single and two step isomerization process for D-tagatose and L-ribose bioproduction using L-arabinose isomerase and D-lyxose isomerase. *Enzyme and Microbial Technology*.

[29] Patel M. J., Patel A. T., Akhani R., Dedania S., Patel D. H. (2016). Bioproduction of d-Tagatose from d-Galactose Using Phosphoglucose Isomerase from Pseudomonas aeruginosa PAO1. *Applied Biochemistry and Biotechnology*.

[30] Shin S., Cao T., Choi J. M. (2017). TM0416, a hyperthermophilic promiscuous nonphosphorylated sugar isomerase, catalyzes various C. *Applied and Environmental Microbiology*.

[31] Jayamuthunagai J., Srisowmeya G., Chakravarthy M., Gautam P. (2017). D-Tagatose production by permeabilized and immobilized Lactobacillus plantarum using whey permeate. *Bioresource Technology*.

[32] Luecke K. J., Bell L. N. (2010). Thermal stability of tagatose in solution. *Journal of Food Science*.

[33] Grant L. D., Bell L. N. (2012). Physical and Chemical Stability of Tagatose Powder. *Journal of Food Science*.

[34] Wu Q., Shah N. P. (2017). The potential of species-specific tagatose-6-phosphate (T6P) pathway in Lactobacillus casei group for galactose reduction in fermented dairy foods. *Food Microbiology*.

[35] Yoshida H., Yoshihara A., Ishii T., Izumori K., Kamitori S. (2016). X-ray structures of the Pseudomonas cichorii D-tagatose 3-epimerase mutant form C66S recognizing deoxy sugars as substrates. *Applied Microbiology and Biotechnology*.

[36] Zheng Z., Mei W., Xia M., He Q., Ouyang J. (2017). X-ray structures of the Pseudomonas cichorii D-tagatose 3-epimerase mutant form C66S recognizing deoxy sugars as substrates. Rational design of Bacillus coagulans NL01 l-arabinose isomerase and use of its F279I variant in d-tagatose production. *Journal of Agricultural and Food Chemistry*.

[37] Oh D.-K. (2007). Tagatose: Properties, applications, and biotechnological processes. *Applied Microbiology and Biotechnology*.

[38] Kim H.-J., Oh D.-K. (2005). Purification and characterization of an L-arabinose isomerase from an isolated strain of Geobacillus thermodenitrificans producing D-tagatose. *Journal of Biotechnology*.

[39] Mei W., Wang L., Zang Y., Zheng Z., Ouyang J. (2016). Characterization of an L-arabinose isomerase from Bacillus coagulans NL01 and its application for D-tagatose production. *BMC Biotechnology*.

[40] Liang M., Chen M., Liu X. (2012). Bioconversion of D-galactose to D-tagatose: Continuous packed bed reaction with an immobilized thermostable L-arabinose isomerase and efficient purification by selective microbial degradation. *Applied Microbiology and Biotechnology*.

[41] Men Y., Zhu Y., Zhang L. (2014). Enzymatic conversion of d-galactose to d-tagatose: Cloning, overexpression and characterization of l-arabinose isomerase from Pediococcus pentosaceus PC-5. *Microbiological Research*.

[42] Zheng Z., Mei W., Xia M., He Q., Ouyang J. (2017). Rational design of Bacillus coagulans NL01 l-arabinose isomerase and use of its F279I variant in d-tagatose production. *Journal of Agricultural and Food Chemistry*.

